# Novel Ternary Heterogeneous Reduction Graphene Oxide (RGO)/BiOCl/TiO_2_ Nanocomposites for Enhanced Adsorption and Visible-Light Induced Photocatalytic Activity toward Organic Contaminants

**DOI:** 10.3390/ma13112529

**Published:** 2020-06-02

**Authors:** Zhanxin Jing, Xiangyi Dai, Xueying Xian, Qiongshan Zhang, Huojiao Zhong, Yong Li

**Affiliations:** College of Chemistry and Environment, Guangdong Ocean University, Zhanjiang 524088, China; 18813497996@163.com (X.D.); xxy970901@163.com (X.X.); 13692360522@163.com (Q.Z.); 15015931358@163.com (H.Z.)

**Keywords:** RGO/BiOCl/TiO_2_, nanocomposites, photocatalyst, visible light

## Abstract

Herein, we describe a simple and cost-effective design for the fabrication of a novel ternary RGO/BiOCl/TiO_2_ nanocomposites through a simple hydrothermal process. The prepared nanocomposites were characterized by Fourier transform infrared spectroscopy (FT-IR), X-ray photoelectron spectroscopy (XPS), scanning electron microscopy (SEM), X-ray diffraction (XRD), UV–vis diffuse reflectance spectroscopy (UV–vis DRS) and N_2_ adsorption–desorption analysis. Organic contaminants—such as methylene blue (MB), methyl orange (MO), rhodamine B (RhB) and amido black-10B (AB-10B)—were employed as the target pollutants to evaluate the adsorption capacity and photocatalytic activity of RGO/BiOCl/TiO_2_ nanocomposites. From experimental data, it was also found that the amount of TiO_2_ impressed the photocatalytic performance, and the nanocomposites with 10% of TiO_2_ showed the best photocatalytic activity. The improved photocatalytic performance may be mainly due to the narrow band gap, and the charge separation and migration of RGO. Moreover, good recyclability was obtained from RGO/BiOCl/TiO_2_ nanocomposites, and scavenger tests indicated that photogenerated holes were the main active species in the reaction system. Therefore, the prepared RGO/BiOCl/TiO_2_ nanocomposites have broad applications foreground in pollutants purification.

## 1. Introduction

With the continuous development of modern industry, organic dyes are widely used in many fields, such as textiles, printing, and dyeing [1]. Wastewater from these related industries is a significant source of water pollution. Over 100,000 commercially available dyes exist, and more than 7 × 10^5^ tons of dyes are produced annually [2]. Although some dyes may not be directly toxic, the dyes in the wastewater undergo chemical and biological changes, consume dissolved oxygen, destroy aquatic life, and endanger human health [3]. With growing demand for environmental purification of hazardous organic pollutants, traditional treatment methods—such as flocculation, adsorption, and biological technology—do not work efficiently because they are either slow or non-destructive to some and most persistent organic pollutants. These methods not only involve expensive equipment, but also have certain limitations in large-scale applications [2,4]. Therefore, it is essential to investigate a novel method to remove toxic compounds from potential sources of water. Recently, “advanced oxidation process” (AOP) is considered as a good alternative to traditional methods because it can non-selectively oxidize multiple organic pollutants [5]. AOP uses the ^•^OH radicals generated by strongly oxidizing substances as the main oxidation species, thereby triggering a series of reactions to break down organic pollutants into smaller and less harmful substances [6]. However, few specific AOP can operate on the natural light sources rather than using artificial light sources [7]. Many studies have been done on the use of metal oxides in water purification, and it can be seen that many metal oxides (such as BiOX (X = Cl, Br, I), TiO_2_, ZnO, and BiVO_4_) can effectively remove organic pollutants through the oxidation process under light [8,9,10,11,12]. However, many metal oxides cannot work under visible light. To develop high-efficiency photocatalysts with visible-light response, nanocomposite technology has been widely used due to its low price and simple preparation [13,14]. Up to now, various nanocomposites as photocatalysts are widely used for the degradation of hazardous organic contaminants in wastewater [15,16,17].

Bismuth-based photocatalytic materials have recently attracted great interest due to their special layered structures [18,19]. For example, BiOCl, which has the internal structure of [Bi_2_O_2_]^2+^ layers interleaved by double slabs of Cl ions, exhibits remarkable photocatalytic property under ultraviolet light irradiation [20]. However, the large indirect band gap (about 3.40 eV) of pure BiOCl leads to its limited photocatalytic activity under sunlight, and the rapid recombination of photogenerated electrons and holes greatly decreases the quantum efficiency [21]. BiOCl-based nanocomposites—such as WO_3_/BiOCl, NaBiO_3_/BiOCl, and BiOCl/TiO_2_—have been reported [22,23,24], and it has been confirmed that BiOCl-based nanocomposites show much higher visible-light photocatalytic activity than pure BiOCl under visible light irradiation, which is attributed to expanding the light absorption region and improving the separation efficiency of photogenerated electrons and holes. TiO_2_, which is non-toxic, stable in aqueous solution, and relatively inexpensive, is a photocatalytically active semiconductor that was widely studied in the early stage [25]. However, when TiO_2_ is used as a photocatalyst, there is a limitation that the charge carriers excited by light cannot be transferred efficiently and are easy to recombine [26]. So far, the composites of TiO_2_ with other semiconductors or conductive materials have been the most common and effective means to solve this problem [27]. Although the method can overcome difficulties in recovering and recycling, it still cannot solve the problem of limited activity and insufficient stability during photocatalytic degradation [28,29], which is due to the rapid recombination of the photogenerated electrons and holes [29]. 

Recently, graphene oxide (GO) and reduced graphene oxide (RGO) have received much attention owing to their high thermal conductivity, large specific surface area and high electron mobility [30,31]. Some studies have demonstrated that the introduction of GO or RGO could contribute to the stronger absorption of visible light, less charge recombination and higher photocatalytic activity of the composites [28,32,33]. Mesoporous TiO_2_-graphene nanocomposites were reported by Liu et al. [34]. The results indicated that the incorporated graphene has a comprehensive effect on the adsorption and charge transfer kinetics of TiO_2_-graphene nanocomposites, which endows the nanocomposites good photocatalytic reactivity and tunable photocatalytic selectivity in decomposing MO and MB in aqueous solution. BiOBr-graphene nanocomposites were prepared by a simple and rapid solvothermal method, found that the resulting nanocomposites exhibited a superior performance on the photocatalytic removal of gaseous nitrogen monoxide than pure BiOBr under visible light irradiation [35]. Herein, we report the simple fabrication of the ternary nanocomposites RGO/BiOCl/TiO_2_ by the solvothermal process. The obtained nanocomposites were characterized by FT-IR, XPS, SEM, XRD, UV–vis diffuse reflectance spectra and N_2_ adsorption–desorption analysis. The prepared ternary nanocomposites exhibit superior photocatalytic activity for the decomposition of several dyes.

## 2. Experimental Section

### 2.1. Materials and Reagents

Bismuth nitrate pentahydrate (Bi(NO_3_)_3_·5H_2_O, AR), titanium tetrachloride (TiCl_4_, AR) and polyvinylpyrrolidone (PVP, *M_n_* = 58,000) were purchased from Shanghai Macklin Biochemical Reagent Co., Ltd., Shanghai, China; potassium chloride (KCl, AR) and ethylene glycol (EG, >99.0%) were provided by Guangdong Guanghua Science and Technology Co., Ltd., Guangzhou, China; graphene oxide (GO) was prepared from graphite according to the modified Hummers method [36], and graphite powder (AR) was purchased from Shanghai Macklin Biochemical Reagent Co. Ltd., Shanghai, China; methylene blue, methyl orange, rhodamine B, and amido black-10B bought from Sinopharm Chemical Reagent in China were used as target degradation products; all other reagents were of analytical grade, and were not purified further. 

### 2.2. Preparation of RGO/BiOCl/TiO_2_ Nanocomposites

Bi(NO_3_)_3_·5H_2_O and TiCl_4_ were first added to 80 mL of ethylene glycol containing 0.2 g of PVP. Then KCl equal to the mole amount of Bi(NO_3_)_3_·5H_2_O was added. After completely dissolved, 50 mg of GO was dispersed in the prepared mixed solution. Afterwards, the prepared dispersion was transferred to a 150 mL Teflon-lined autoclave, and placed at 180 °C for 8 h. After the reaction, the product was collected by centrifugation, and washed three times with ethanol and distilled water. To prepare RGO/BiOCl/TiO_2_ nanocomposites with different TiO_2_ contents, the percentage of TiCl_4_ is set to 0%, 10%, 30%, and 50% with respect to the sum amount (20 mmol) of Bi(NO_3_)_3_·5H_2_O and TiCl_4_, respectively. The resulting sample was marked as RGO/BiOCl/TiO_2_-x%, where x% represents the theoretical content of TiO_2_ to the total amount of BiOCl and TiO_2_ in the nanocomposites. 

### 2.3. Characterization

Fourier transform infrared spectroscopy (FT-IR) of the prepared nanocomposites was performed by a Nicolet-460 spectrometer (Thermo Electron Scientific Instruments, Waltham, MA, USA) in the range of 4000–400 cm^−1^. The X-ray photoelectron spectra (Thermo Fisher Scientific (SID-Elemental), Waltham, MA, USA) were recorded using Al K_α_ radiation. The morphologies of the samples were observed by a scanning electron microscopy (SEM, ZEISS SUPRA 40, Jena, Germany). The crystalline structures of the nanocomposites were analyzed by X-ray diffraction (XRD) using a Bruck D8 Advance diffractometer (Hitachi Inc., Krefeld, Germany) at a rate of 10°/min. UV–vis diffuse reflectance spectra were recorded in a Shimadzu Corporation UV-270 spectrophotometer (Kyoto, Japan). The Brunauer–Emmett–Teller (BET) N_2_ adsorption–desorption analysis was conducted on a Micro for Tri Star II Plus 2.02 gas sorption analyzer (Micromeritics Instrument Corp, Norcross, GA, USA). The pore-size distributions of the samples were also estimated using the Barrett–Joyner–Halenda (BJH) method.

### 2.4. Adsorption and Photocatalytic Performance Measurement

The adsorption and photocatalytic capacities of the as-prepared nanocomposites were evaluated in the XRA-7CG4 photo-reaction apparatus (Nanjing Xujiang Machinery Power Factory, Nanjing, China). The adsorption capacity of the prepared nanocomposites was measured by the following description. The prepared nanocomposites were first added to the quartz test tubes, and 10 mL of the solution containing organic pollutants was added. Afterwards, the quartz test tubes were placed into the photo-reaction apparatus, and magnetically stirred in the dark. After a certain time, the test tube was taken out. The mixture was separated by centrifugation at 16,000 r/min for 3 min, and the concentration of the obtained supernatant was measured by using a UV–visible spectrophotometer (UV2800S, Shanghai Shunyu Hengping Scientific Instrument Co., Ltd., Shanghai, China). The amount of organic pollutants adsorbed by the prepared nanocomposites was also calculated.

Photocatalytic property of the prepared nanocomposites was evaluated by the following method. A certain weight of the nanocomposites was added to the quartz test tubes with 10 mL of organic pollutant solution (30 mg/L). Prior to illumination, the solution was magnetically stirred for 30 min in the dark to reach the adsorption equilibrium between the nanocomposites and organic pollutants. Then, the solution was exposed to a 500W mercury lamp with the cut-off filter (λ > 400 nm). After irradiating for a certain period of time, a tube was removed and the suspension was centrifuged to remove the nanocomposites. Finally, the supernatant was removed and the concentration of the remaining organic pollutants in the solution was measured with a UV–visible spectrophotometer (UV2800S, Shanghai Shunyu Hengping Scientific Instrument Co., Ltd., Shanghai, China).

### 2.5. Analysis of Photocatalytic Mechanism

For analyzing the active species during photocatalytic process, hydroxyl radicals (^•^OH), superoxide radicals (O_2_^•−^), photogenerated electrons (e^−^), and holes (h^+^) were investigated by adding isopropanol (IPA), 1,4-benzoquinone (BQ), tert-butanol (TBA), and ethylenediaminetetraacetic acid disodium salt (EDTA), respectively [37,38]. The method was similar to the photocatalytic degradation test previously described except that 1.0 mmol of scavenger was added.

## 3. Results and Discussions

### 3.1. Chemical Structure of RGO/BiOCl/TiO_2_ Nanocomposites

**Figure 1:**
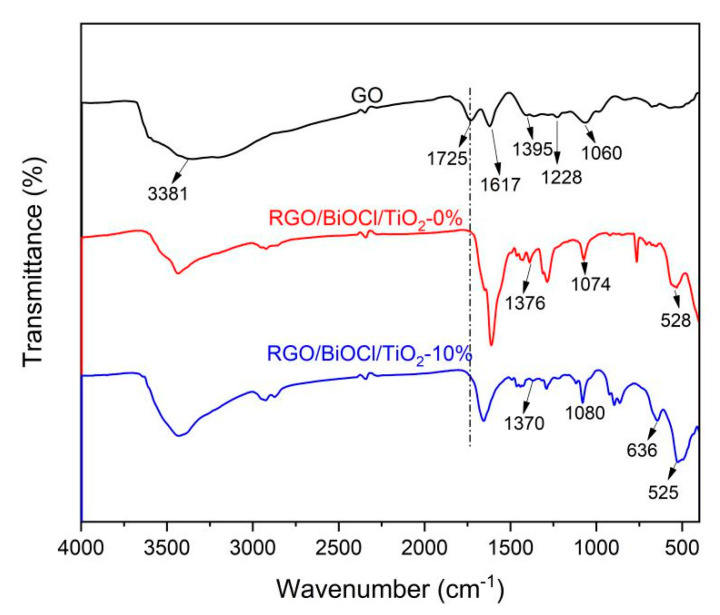
FT-IR spectra of GO and RGO/BiOCl/TiO_2_ nanocomposites with different TiO_2_ contents.

Figure 1 displays the FT-IR spectra of GO and RGO/BiOCl/TiO_2_ nanocomposites with various TiO_2_ contents. In FT-IR spectrum of GO, there exhibits several obvious absorption peaks at 3381, 1725, 1395, 1228, and 1060 cm^−1^, which are assigned to the O-H stretching vibration of hydroxy groups, carboxyl or carbonyl stretching vibration, O-H deformation in the C-OH groups, C-OH stretching vibration and C-O stretching vibration in C-O-C in epoxide, respectively [39,40]. This indicates that there are oxidation functional groups on the graphite surface after oxidation process. The peak at 1617 cm^−1^ is attributed to the skeletal vibration of graphene. It is clear that for the RGO/BiOCl/TiO_2_ nanocomposites, the peak at 1725 cm^−1^ corresponding to C=O stretching vibration observed on GO disappears, revealing that GO has been reduced to RGO during hydrothermal process [41]. For RGO/BiOCl/TiO_2_ nanocomposites without TiO_2_, the peak at 528 cm^−1^ is attributed to the Bi-O stretching vibration, which is the characteristic peak of BiOCl [42]. The peaks at 1376 and 1074 cm^−1^ may be attributed to asymmetric and symmetric stretching vibrations of Bi-Cl. Compared with RGO/BiOCl/TiO_2_ nanocomposites without TiO_2_, there is a clear peak at 636 cm^−1^ in the FT-IR spectrum of RGO/BiOCl/TiO_2_ nanocomposites with 10% of TiO_2_, which may be assigned to the Ti-O bond vibration in TiO_2_.

**Figure 2:**
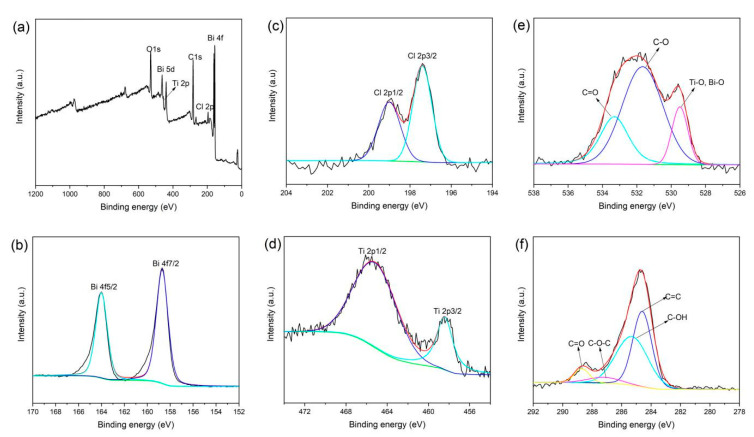
(**a**) XPS wide scan spectrum of RGO/BiOCl/TiO_2_ nanocomposites; (**b**–**f**) XPS high resolution spectra of RGO/BiOCl/TiO_2_ nanocomposites: (**b**) Bi 4f, (**c**) Cl 2p, (**d**) Ti 2p, (**e**) O1s, and (**f**) C 1s.

Further, XPS was used to analyze the surface chemical states of RGO/BiOCl/TiO_2_ nanocomposites. As displayed in Figure 2a, the survey spectrum of the nanocomposites with 10% of TiO_2_ clearly shows the signals of C, O, Bi, Cl, and Ti. The Bi 4f7/2 and 4f5/2 peaks are observed at 158.7 and 164.0 eV, respectively, indicating that the Bi^3+^ is the dominant state in the RGO/BiOCl/TiO_2_ nanocomposites [18,24], as showed in Figure 2b. The high-resolution Cl 2p spectrum in Figure 2c shows two obvious peaks at 197.4 and 198.9 eV, which can be ascribed to Cl 2p3/2 and Cl 2p1/2, respectively. In terms of the Ti 2p spectrum (Figure 2d), the binding energies at 458.4 and 465.6 eV correspond to Ti 2p3/2 and 2p1/2, respectively. The O 1s spectrum in Figure 2e can be fitted into three different peaks at 529.4, 531.7, and 533.4 eV, the peak of O1s at 529.4 eV should be ascribed to the Ti-O and Bi-O bonds [43]. In addition, the peaks at 533.4 and 531.7 eV are attributed to oxygen bonded to the carbon (C=O, C-O). In Figure 2f, the peaks for C 1s signals of the RGO/BiOCl/TiO_2_ nanocomposites were deconvoluted into four signals at 284.6, 285.4, 287.3, and 288.8 eV, which are attributed to C=C, C-OH, C-O-C, and C=O, respectively. Compared with GO (Figure S1), it can be observed that the percentage of these oxygen-containing functional groups in the RGO/BiOCl/TiO_2_ nanocomposites obviously decreases, indicating that partial GO is reduced to graphene during hydrothermal reaction. This is consistent with the results of FT-IR. Therefore, RGO/BiOCl/TiO_2_ nanocomposites were successfully prepared through a simple solvothermal process.

### 3.2. Morphology of RGO/BiOCl/TiO_2_ Nanocomposites

**Figure 3:**
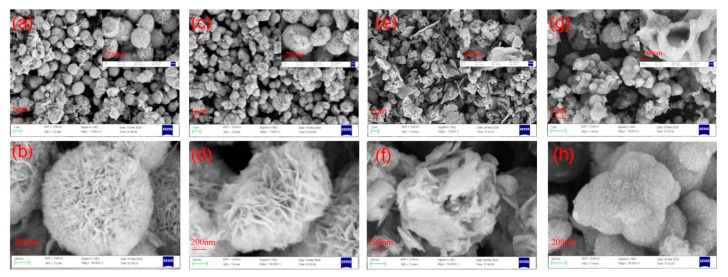
SEM images of RGO/BiOCl/TiO_2_ nanocomposites with various TiO_2_ contents: (**a**,**b**) 0%; (**c**,**d**) 10%; (**e**,**f**) 30%; and (**g**,**h**) 50%.

SEM images of GO and RGO/BiOCl/TiO_2_ nanocomposites with various compositions are exhibited in Figure S2 and Figure 3, respectively. As Figure S2, there clearly shows the sheet structure of GO, and the typical wrinkle structures are also observed on GO nanosheets, which could be attributed to both the intrinsic (thermal fluctuation) and extrinsic (defects, functionalization, and applied stresses) factors [44,45]. The RGO/BiOCl/TiO_2_ nanocomposites without TiO_2_ displayed a regular flower-like 3D structure with about 1µm of particle size (as Figure 3a,b). When TiO_2_ was introduced during the synthesis process, the obtained RGO/BiOCl/TiO_2_ nanocomposites showed an uneven flower-like 3D structure, and their surfaces became rough, as shown in Figure 3c–f. It can be observed that 3D microspheres are constructed by the assembly nanoflakes when the TiO_2_ content is low (≤30%). Subsequently, with the further increase of the TiO_2_ content, the flower-like 3D structure disappears, as Figure 3g,h. The nanocomposite with 50% of TiO_2_ forms a raspberry-like structure, and its surface shows very small pore structures, which is significantly different from the nanocomposites with low TiO_2_ content (≤30%). Whereas no obvious structure of RGO is observed, the possible reason is that RGO is embedded in the self-assembly of nanosheets.

**Figure 4:**
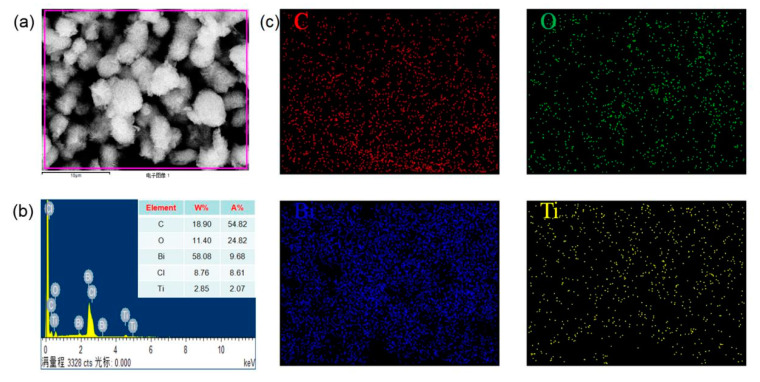
(**a**) Representative SEM image, (**b**) EDX spectrum, and (**c**) elemental mapping of RGO/BiOCl/TiO_2_ nanocomposites with 10% of TiO_2_.

The chemical component of the typical morphology (as Figure 4a) RGO/BiOCl/TiO_2_ nanocomposites with 10% of TiO_2_ was analyzed by EDX. The EDX pattern of RGO/BiOCl/TiO_2_ nanocomposites in Figure 4b verifies that the prepared nanocomposites are composed of C, O, Bi, Cl, and Ti elements, which is consistent with the XPS results. It can be found that the atomic ratio of Ti (A% = 2.07%) and Bi (A% = 9.68%) is about 19: 90, which is significantly higher than the theoretical atomic ratio of Ti and Bi. The corresponding elemental mapping images in Figure 4c show that these elements are homogeneously distributed, revealing that the RGO nanosheets, TiO_2_ and BiOCl nanoparticles are fully contacted and well-assembled. This is beneficial to the formation of heterojunctions between the components in the nanocomposites. These results also demonstrate the high purity of the prepared RGO/BiOCl/TiO_2_ nanocomposites.

### 3.3. Phase Structure and Optical Property

**Figure 5:**
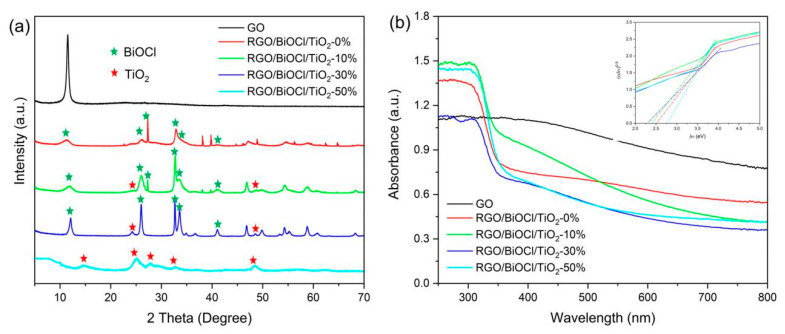
(**a**) XRD patterns and (**b**) UV–vis diffusive reflectance spectra of as-prepared samples.

Figure 5a shows the XRD patterns of GO and RGO/BiOCl/TiO_2_ nanocomposites with different TiO_2_ contents. The XRD pattern of GO reveals the characteristic reflection peak at 2θ = 11.6°, which is assigned to a d-spacing of 0.83 nm in the lamellar structure of GO [46]. For RGO/BiOCl/TiO_2_ nanocomposites without TiO_2_, the diffraction peaks at 2θ ≈ 12.0°, 26.1°, 27.3°, 32.8°, 33.6°, and 41.4° can be readily assigned to the (001), (002), (101), (110), (102), and (112) planes of BiOCl (JCPDS 06-0249), respectively. The diffraction peak appearing at 10.4° for GO obviously reduces, which may be attributed to the reduction of GO. With the increase of the TiO_2_ content, some diffraction peaks would broaden to some extent, and the diffraction peak at 27.3° corresponding to the (101) plane of BiOCl gradually disappears, and the diffraction peaks at 25.0° and 48.6° corresponding to TiO_2_ gradually appear, revealing the coexistence of BiOCl and TiO_2_ [43,47]. These results reveal that BiOCl/TiO_2_ binary oxides are loaded on the surface of RGO sheets. When the TiO_2_ content reaches 50%, there exhibits several weak diffraction peaks. The peak at 25.0° is due to (101) plane of anatase TiO_2_ (JCPDS 21-1272), while the peaks at 2θ = 14.7°, 27.9°, 32.8°, and 48.6° are attributed to (200), (002), (311), and (601) crystal planes of TiO_2_ (JCPDS 46-1238) [47]. However, the diffraction peaks of BiOCl are not observed, and the RGO/BiOCl/TiO_2_ nanocomposites with 50% of TiO_2_ exhibit low crystallinity. This may be due to the mutual interference during the growth of different crystals, leading to a large number of defects in the crystals, which reduces the crystal size and crystallinity.

UV–vis diffusive reflectance spectra of GO and RGO/BiOCl/TiO_2_ nanocomposites with different TiO_2_ contents are displayed in Figure 5b. It is clear that GO exhibits excellent light absorption capacity in the range of the visible region, which is attributed to its color. The prepared RGO/BiOCl/TiO_2_ nanocomposites also exhibit obvious absorption in the visible light range. This can be attributed to the 3D flower-like structures assembled by nanosheets, allowing the light to multi-reflection [48]. The absorption capacity of the prepared nanocomposites in the visible light range is closely related to their chemical compositions, which leads to different morphologies. This has been confirmed by SEM. As a crystalline semiconductor, the absorption near the band edge follows the formula [37,48]

(1) $${\left(\alpha hv\right)}^{n}=k(hv-E_{g})$$

where α, *hv*, *k*, and *E_g_* are absorption coefficient, absorption energy, absorption constant, and band gap, respectively. The index n is equal to 2 for direct transition and 0.5 for the indirect transition. As a result, the Eg can be estimated from a plot of the (α*hv*)^0.5^ *vs hv* plots as shown in the illustration in Figure 5b. The estimated band gaps of the prepared RGO/BiOCl/TiO_2_ nanocomposites are about 2.30–2.77 eV (as listed in Table 1). It is clear that the estimated band gaps of the nanocomposites first decrease and then increase, and the nanocomposites with 30% of TiO_2_ have the smallest band gap. Compared with the estimated band gaps (about 3 eV) of TiO_2_ and BiOCl reported in the literature [37,48], the RGO/BiOCl/TiO_2_ nanocomposites present the narrowest band gap, revealing that they could be easily excited by visible light. The enhanced optical activity of the prepared nanocomposites can be attributed to the heterojunction structures formed in the nanocomposites, causing a band gap red shift. This ensures efficient harvesting of visible light irradiation, which substantiates its potentiality in visible-light-driven photocatalysis.

### 3.4. Surface Area and Pore-Size Distribution

**Figure 6:**
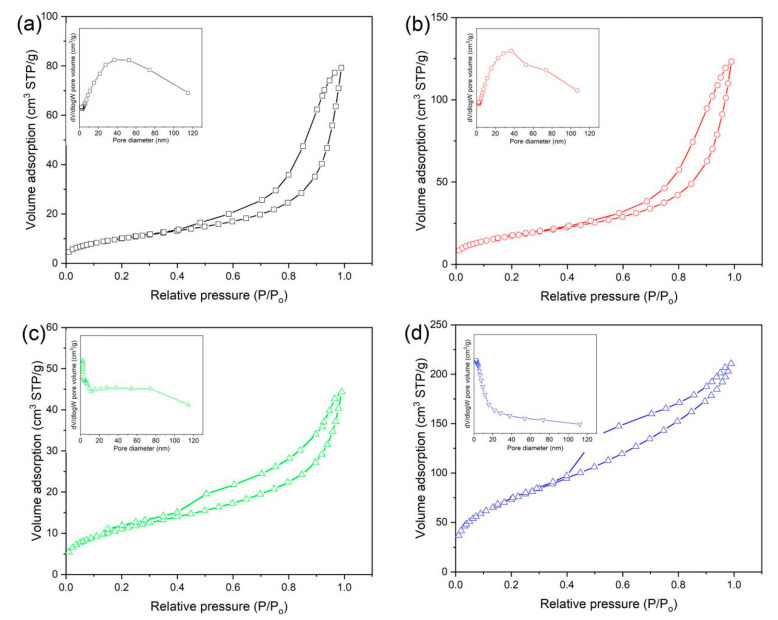
Nitrogen adsorption–desorption isotherm and the corresponding pore size distribution plots of RGO/BiOCl/TiO_2_ nanocomposites with various TiO_2_ contents: (**a**) 0%; (**b**) 10%; (**c**) 30%; and (**d**) 50%.

Figure 6 shows the N_2_ adsorption–desorption isotherm of RGO/BiOCl/TiO_2_ nanocomposites with various compositions and their corresponding pore-size distribution. It is clear that all of the samples exhibit a type IV isotherm with a hysteresis loop within the range from 0.4 to 1.0 (P/P_0_), indicating their mesoporous nature according to the IUPAC-BET classification [49,50]. The Barrett–Joyner–Halenda pore-size distribution of the prepared nanocomposites is shown in the inset of the corresponding N_2_ adsorption–desorption isotherm, founding that the pore diameter of the prepared nanocomposites is mainly distributed in mesoporous range of 2–40 nm. This further demonstrates the mesoporous nature of the prepared nanocomposites. The BET surface area, pore volume and average pore size of the prepared nanocomposites are listed in Table 1. For RGO/BiOCl/TiO_2_ nanocomposites without TiO_2_, its BET surface area, pore volume and average pore size are 37.58 m^2^/g, 0.121 cm^3^/g, and 13.05 nm, respectively. With the introduction of TiO_2_, the BET properties of the prepared nanocomposites have changed significantly. At low TiO_2_ content (≤30%), the BET surface area first increases and then decreases as the TiO_2_ content increases, and the nanocomposites with 10% of TiO_2_ exhibit large BET surface area (64.21 m^2^/g). However, when the content of TiO_2_ is 50%, BET surface area of the nanocomposites increases rapidly, reaching 270.59 m^2^/g. This is because the nanocomposites with various compositions have different surface morphologies, which have been confirmed by SEM. It can also be observed from Table 1 that the average pore size of the nanocomposites decreases from 13.05 nm to 5.27 nm as the content of TiO_2_ increases from 0% to 50%. Generally, the larger the surface area of the material, the higher its adsorption capacity due to the increase of active sites. While the pore structure is also a factor that limits the adsorption of the pollutants on the nanocomposites, the larger the pore structure of the nanocomposites, the more pollutants will be adsorbed on its surface [51]. Therefore, the adsorption behavior of the nanocomposites should be studied to deeply analyze their microstructures. 

### 3.5. Adsorption Capacity

**Figure 7:**
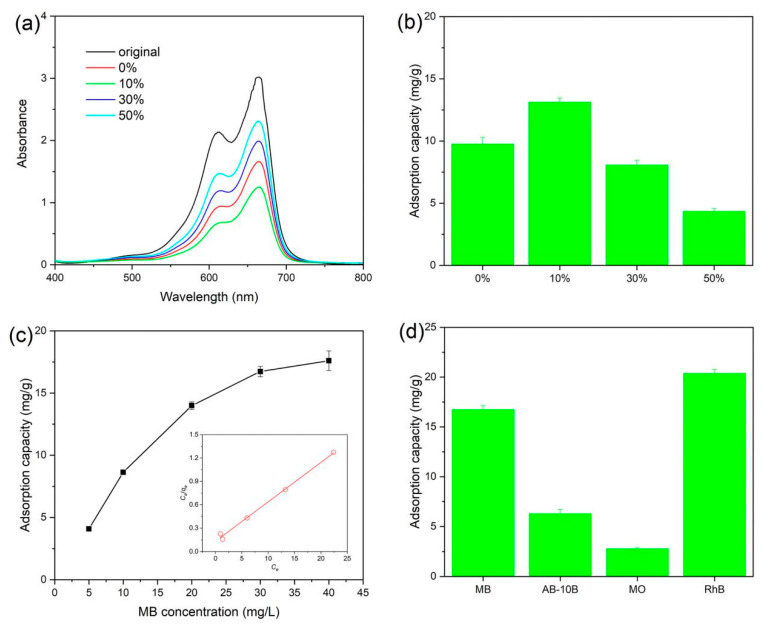
Adsorption capacity of RGO/BiOCl/TiO_2_ nanocomposites: (**a**) UV–vis absorption spectra of MB dye adsorption by RGO/BiOCl/TiO_2_ nanocomposites; (**b**) adsorption capacity of different RGO/BiOCl/TiO_2_ nanocomposites for MB (MB concentration: 20 mg/L; the amount of the nanocomposites: 1.0 g/L; time: 30 min); (**c**) effect of MB concentration on adsorption capacity (the amount of RGO/BiOCl/TiO_2_-10% nanocomposites: 1.0 g/L; 30 min) and the corresponding Freundlich isotherm; (**d**) adsorption capacity of RGO/BiOCl/TiO_2_-10% nanocomposites for different dyes (dye concentration: 30 mg/L; the amount of the nanocomposites: 1.0 g/L; time: 30 min).

Figure 7 displays the adsorption property of RGO/BiOCl/TiO_2_ nanocomposites. Adsorption behavior of RGO/BiOCl/TiO_2_ nanocomposites with various compositions is shown in Figure 7a. The major peak at the absorption spectra of MB solution occurs at 664 nm, and the RGO/BiOCl/TiO_2_ nanocomposites with 10% of TiO_2_ show the most significant reduction in absorption intensity of MB solution. Furthermore, the adsorption capacity of MB on the RGO/BiOCl/TiO_2_ nanocomposites calculated from the absorption spectra of the MB solution is presented in Figure 7b. It can be observed that the adsorption capacity of MB on the nanocomposites is closely related to the composition. As the content of TiO_2_ increases, the adsorption capacity increases first and then decreases. The adsorption amount of the RGO/BiOCl/TiO_2_ nanocomposites with 10% of TiO_2_ is the largest, which can reach 13.1 mg/g. This reveals the adsorption capacity of the nanocomposites is determined by the specific surface area and pore size, and the large specific surface area and suitable pore structure can give the nanocomposites a good adsorption capacity. Although the nanocomposites with 50% of TiO_2_ have a large specific surface area, the small pore size limits their adsorption to MB. The influence of MB concentration on adsorption behavior of the nanocomposites RGO/BiOCl/TiO_2_-10% is displayed in Figure 7c. As MB concentration increases from 5 mg/L to 40 mg/L, the amount of MB adsorbed on the nanocomposites increases from 4.1 mg/g to 17.6 mg/g. It can be found that when the concentration of MB is relatively large (>30 mg/L), the adsorption capacity of MB on the nanocomposites increases slowly. The increased MB concentration promotes the rapid binding of MB molecules to the adsorption sites of RGO/BiOCl/TiO_2_ nanocomposites, and accelerates the saturation of the adsorption sites on the surface of the nanocomposites. When the MB concentration is large, the adsorption sites on the surface of RGO/BiOCl/TiO_2_ nanocomposites are close to saturation. Therefore, the adsorption amount of MB on RGO/BiOCl/TiO_2_ nanocomposites does not increase significantly as the MB concentration further increases. It is also observed in the illustration in Figure 7c that the adsorption process follows the Langmuir adsorption isotherm with R^2^ = 0.993, indicating that MB molecules were adsorbed on the prepared nanocomposites by a monolayer adsorption [52]. The adsorption of different dyes on RGO/BiOCl/TiO_2_ nanocomposites is also discussed, and the obtained results are shown in Figure 7d. It is clear that the adsorption amount of different dyes on the nanocomposites is different, and follows the following sequence: RhB > MB > AB-10B > MO. For RhB, its adsorption on the nanocomposites can reach 20.3 mg/g. The possible reason is that the structural differences of different dyes could lead to different interaction forces between the nanocomposites and the dyes. The high adsorption capacity of the nanocomposite may improve its photocatalytic activity during the photocatalytic reaction.

### 3.6. Photocatalytic Performance

**Figure 8:**
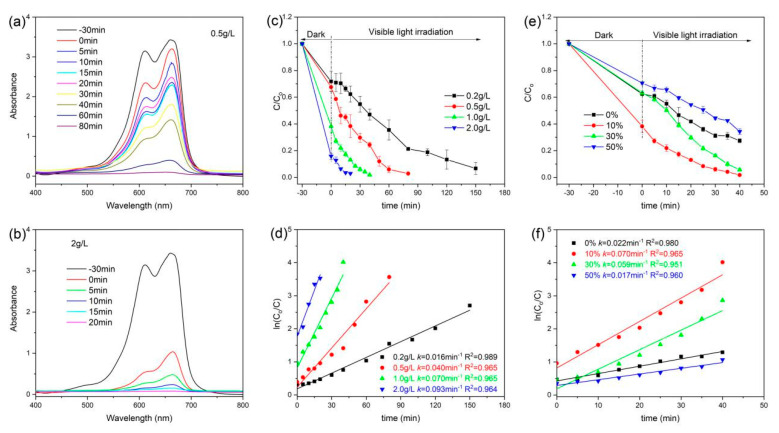
(**a**,**b**) UV–vis absorption spectra of MB dye degradation by RGO/BiOCl/TiO_2_-10% nanocomposites under visible light irradiation; (**c**) photodegradation efficiency of MB dye under various nanocomposite concentrations; (**d**) the corresponding fit curves obtained by pseudo-first-order kinetic model; (**e**) photodegradation efficiency of MB dye under the nanocomposites with various TiO_2_ contents (the nanocomposite concentration: 1.0 g/L); and (**f**) the corresponding fit curves obtained by pseudo-first-order kinetic model.

The photocatalytic activity of the prepared RGO/BiOCl/TiO_2_ nanocomposites for organic contaminants was investigated. As displayed in Figure 8a,b, the absorption peak intensity of MB dye at 664 nm is rapidly weakening as the irradiation time increases. When the amount of RGO/BiOCl/TiO_2_ nanocomposites is 0.5 g/L, the absorption peak of MB dye disappears after 80 min of irradiation. For MB solution with 2.0 g/L of RGO/BiOCl/TiO_2_ nanocomposites, the adsorption peak of MB dye can completely disappear after 20 min of irradiation (as Figure 8b). The degradation efficiency calculated from the absorption spectra is shown in Figure 8c. It is clear that photodegradation efficiency obviously increases as the amount of RGO/BiOCl/TiO_2_ nanocomposites increases. When the amount of RGO/BiOCl/TiO_2_ nanocomposites was 0.2, 0.5, 1.0, and 2.0 g/L, the apparent degradation rate constants of MB dye obtained by the pseudo-first-order kinetic model were 0.016, 0.040, 0.070, and 0.093 min^−1^, respectively (as Figure 8d). It is clear that the degradation rate increases as a reduced amplitude with increasing the amount of the nanocomposites. Therefore, in order to save the nanocomposites, the amount of RGO/BiOCl/TiO_2_ nanocomposites was chosen to be 1.0 g/L in the subsequent experiments.

To further investigate the effect of the nanocomposite composition on the photocatalytic activity, the RGO/BiOCl/TiO_2_ nanocomposites with different compositions were also performed for MB dye degradation, and the results are shown in Figure 8e. In the photocatalytic system of RGO/BiOCl/TiO_2_ nanocomposites without TiO_2_, 72.5% of MB dye is removed after 40 min of irradiation. With increasing of the TiO_2_ content in the nanocomposites, the photocatalytic efficiency of the RGO/BiOCl/TiO_2_ nanocomposites for MB dye first increases and then decreases. The RGO/BiOCl/TiO_2_ nanocomposites with 10% of TiO_2_ exhibit the highest photodegraded efficiency, degrading nearly 98.2% of MB dye after 40 min of irradiation. It is also found that MB dye degradation over RGO/BiOCl/TiO_2_ nanocomposites with different compositions fits the pseudo-first-order kinetic model, as displayed in Figure 8f. It is clear that the apparent degradation rate constants of RGO/BiOCl/TiO_2_ nanocomposites first increase and then decrease as the TiO_2_ content increases. For the nanocomposites RGO/BiOCl/TiO_2_-10%, the apparent degradation rate constant attains a maximum value of 0.070 min^−1^, which is 3.18 times higher than that of RGO/BiOCl/TiO_2_ nanocomposites without TiO_2_ (0.022 min^−1^). The high adsorption capacity of RGO/BiOCl/TiO_2_-10% can contribute to the improvement in photocatalytic activity, and the lower TiO_2_ content is conducive to the formation of heterojunctions in the nanocomposite, which could improve the separation and migration efficiency of the electron–hole pairs. Therefore, the photocatalytic activity of the nanocomposites RGO/BiOCl/TiO_2_-10% is obviously improved.

**Figure 9:**
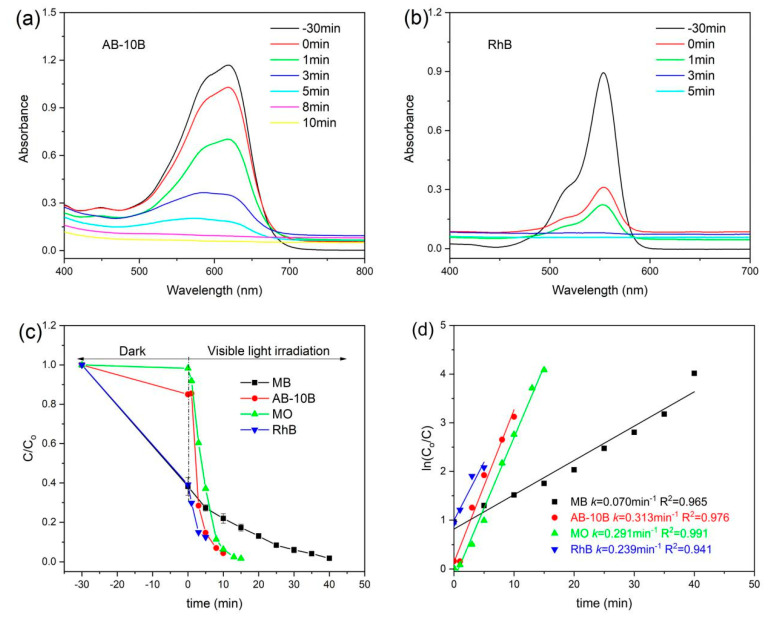
UV–vis absorption spectra of (**a**) AB-10B and (**b**) RhB dyes degradation by RGO/BiOCl/TiO_2_ nanocomposites with 10% of TiO_2_ under visible light irradiation (the nanocomposite concentration: 1.0 g/L); (**c**) photodegradation efficiency of different dyes over RGO/BiOCl/TiO_2_ nanocomposites and (**d**) the corresponding fit curves obtained by pseudo-first-order kinetic model.

To determine the feasibility of the RGO/BiOCl/TiO_2_ nanocomposites, the photocatalytic activity was further tested by analyzing the photodegradation behaviors of MB, AB-10B, MO, and RhB under visible light. As showed in Figure 9a,b, with the irradiation of visible light, the maximum absorption peaks of various dyes rapidly decrease. The degradation efficiency calculated from the absorption spectra is displayed in Figure 9c. It is clear that in a short irradiation time, the removal efficiencies of various dyes by the RGO/BiOCl/TiO_2_ nanocomposites can reach more than 95%. The photocatalytic process of RGO/BiOCl/TiO_2_ nanocomposites for the four dyes follows the pseudo-first order kinetic model, as Figure 9d. It is clear that the nanocomposites have high rate constant (0.239–0.313 min^−1^) for the degradation of AB-10B, MO, and RhB, which is 3.40–4.47 times higher than that of MB degradation of the nanocomposites (0.070 min^−1^). This may be due to the difference in the molecular structure of the dyes. However, it is different from the adsorption behavior of dyes on the nanocomposites, revealing that the adsorption capacity of the RGO/BiOCl/TiO_2_ nanocomposites is not the determinant of their photocatalytic performance. The excellent photocatalytic performance of the nanocomposites may be attributed to the narrow band gap, promoting the separation of the electron–hole pairs. Compared with the existing literature reports of TiO_2_, BiOCl, BiOCl/TiO_2_, RGO/TiO_2_, and RGO/BiOCl [37,43,46,53], the prepared RGO/BiOCl/TiO_2_ nanocomposites show higher activity of degradation for various organic dyes. These photodegradation results of four model organic dyes demonstrate that RGO/BiOCl/TiO_2_ nanocomposites are efficient visible-light-driven photocatalysts, which exhibits broad spectrum photocatalytic degradation activity. Therefore, the significant activity of RGO/BiOCl/TiO_2_ nanocomposites may let them become valuable photocatalytic materials in potential applications for environmental protection.

To show the advantage of RGO/BiOCl/TiO_2_ nanocomposites, the obtained results in this study have been compared with some reported catalysts in the literature [14,22,29,37,43], as summarized in Table 2. It is clear that the most reported methods require long reaction time or low dye concentration. The nanocomposites synthesized in this study can achieve high removal ratio at high dye concentration and short reaction time, so the present method is more suitable and superior.

### 3.7. Reusability Study

**Figure 10:**
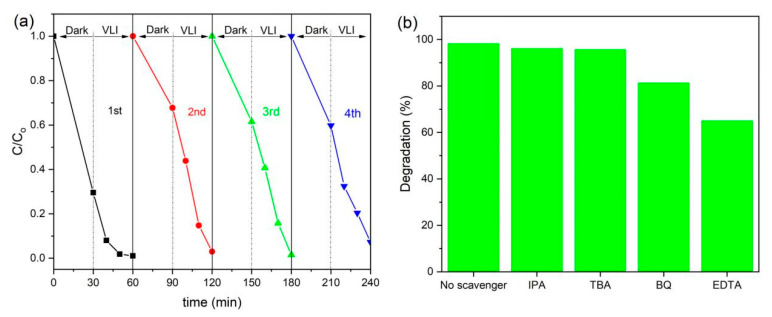
(**a**) Reusability test of RGO/BiOCl/TiO_2_ nanocomposites for the photodegradation of MB; (**b**) Effects of different scavengers on degradation of MB in the presence of RGO/BiOCl/TiO_2_ nanocomposites under visible light irradiation.

Reusability is a very important factor for determining the use of the photocatalysts in practical applications. To investigate the photocatalytic stability of RGO/BiOCl/TiO_2_ nanocomposites, four reaction cycles were performed, and after each reaction, the nanocomposites were collected by centrifugal separation (16,000 r/min), and washed three times with distilled water and ethanol. The collected nanocomposites were freeze-dried before proceeding to the next photocatalytic reaction. As shown in Figure 10a, only about 4% reduction in photocatalytic efficiency of RGO/BiOCl/TiO_2_ nanocomposites is observed after four cycles. This reveals that the synthesized RGO/BiOCl/TiO_2_ nanocomposites are reusable and recyclable photocatalysts in environmental applications.

### 3.8. Photocatalytic Mechanism

The use of photocatalytic technology to decompose organic dyes is a redox process, in which many reaction intermediates are produced, such as the hydroxyl radicals (^•^OH), the holes (h^+^), the superoxide radicals (O_2_^•−^), and photogenerated electrons (e^−^). To analyze the mechanism of the synthesized RGO/BiOCl/TiO_2_ nanocomposites for catalytic degradation of organic dyes, IPA, TBA, BQ, and EDTA were used as scavengers for ^•^OH, e^−^, O_2_^•−^, and h^+^, respectively [37,38]. As displayed in Figure 10b, when IPA or TBA is added in the degradation system, the degradation efficiency will be slightly reduced, which is 96.1% and 95.6%, respectively. This indicates that ^•^OH and e^−^ are not the main active substances in the process of RGO/BiOCl/TiO_2_ nanocomposites catalyzing degradation of MB under visible light. When BQ was added in the above degradation system, the efficiency of RGO/BiOCl/TiO_2_ nanocomposites to degrade MB under visible light decreased to 81.2%, indicating that O_2_^•−^ is involved in the nanocomposites catalyzing MB degradation process under visible light. In particular, when EDTA was added in the above photodegradation system, a significant effect can be observed, indicating that the holes (h^+^) play an important role in the visible light degradation of MB catalyzed by RGO/BiOCl/TiO_2_ nanocomposites, which are the main active species. Based on these results, the possible mechanism for the visible-light-catalyzed degradation of MB by the prepared RGO/BiOCl/TiO_2_ nanocomposites is proposed as

(2) $$RGO/B\mathrm{iO}Cl/T{\mathrm{iO}}_{2}+\mathrm{hv}\to RGO/B\mathrm{iO}Cl/T{\mathrm{iO}}_{2}^{}(h^{+}+e^{-})$$

(3) $$O_{2}+e^{-}\to O_{2}^{•-}$$

(4) $$MB+O_{2}^{•-}\to \cdots \to \mathrm{degradation}\;\mathrm{products}$$

(5) $$MB+h^{+}\to \cdots \to \mathrm{degradation}\;\mathrm{products}$$

**Figure 11:**
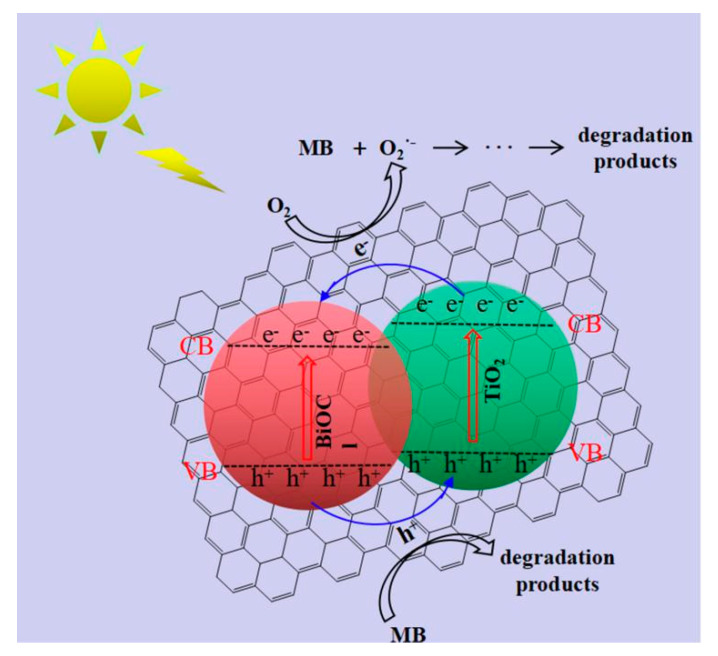
Proposed mechanism of photocatalytic activity by RGO/BiOCl/TiO_2_ nanocomposites.

Based on these results, a mechanism for the photocatalytic activity over the prepared RGO/BiClO/TiO_2_ nanocomposites under visible light is proposed, as shown in Figure 11. When the RGO/BiOCl/TiO_2_ nanocomposites are irradiated by visible light, it is accompanied by the generation of e^−^ and h^+^ (Equation (2)). Due to the existence of graphene or the possibility that BiOCl/TiO_2_ has formed a heterojunction, e^−^ would be rapidly transferred from the CB of TiO_2_ to the CB of BiOCl. During this process, O_2_ molecules at the interface would react with e^−^ to produce O_2_^•−^ active radicals (Equation (3)), whose exitence has been demonstrated during the photodegradation process by adding BQ as a scavenger. The formed O_2_^•−^ could promote the degradation of MB (Equation (4)). In addition, the photogenerated h^+^ on the VB of BiOCl will be rapidly transferred to the VB of TiO_2_ through the interface of the heterojunctions, and react with MB to CO_2_, H_2_O, and degraded products (Equation (5)). The excellent photocatalytic activity of RGO/BiOCl/TiO_2_ nanocomposites is attributed to low band gap caused by the heterojunction structures in the nanocomposites, which accelerates the separation of the electron–hole pairs [54]. In addition, RGO as an electron mediator can also contribute to the effective separation and migration of charges. These enhance the photocatalytic activity of RGO/BiOCl/TiO_2_ nanocomposites.

## 4. Conclusions

In summary, RGO/BiOCl/TiO_2_ nanocomposites with highly photocatalytic activity for organic dyes were prepared through a simple hydrothermal process. The RGO/BiOCl/TiO_2_ nanocomposites exhibited higher photocatalytic performance to MB dye under visible light irradiation, and the nanocomposites with 10% of TiO_2_ showed the highest photocatalytic activity. Furthermore, the prepared nanocomposites also exhibited almost complete degradation of MO, RhB, and AB-10B dyes under visible light irradiation. The obviously enhanced photocatalytic activity of the prepared nanocomposites was attributed to narrow band gap and RGO being an electron mediator for effective charge separation and migration. Meanwhile, a possible photocatalytic mechanism based on the prepared RGO/BiOCl/TiO_2_ nanocomposites has been proposed. Recyclable RGO/BiOCl/TiO_2_ nanocomposites, as highly efficient photocatalysts, have a large potential for removing organic pollutants from wastewater.

## Figures and Tables

**Table 1:** Physicochemical characteristics of the prepared nanocomposites

Nanocomposites	BET Surface Area (m^2^/g)	Pore Volume (cm^3^/g)	Average Pore Size (nm)	Band Gap (eV)
RGO/BiOCl/TiO_2_-0%	37.58	0.121	13.05	2.51
RGO/BiOCl/TiO_2_-10%	64.21	0.188	12.52	2.39
RGO/BiOCl/TiO_2_-30%	40.44	0.069	7.60	2.30
RGO/BiOCl/TiO_2_-50%	270.59	0.331	5.27	2.77

**Table 2:** Comparison of catalytic activity of RGO/BiOCl/TiO_2_ nanocomposites with some reported catalysts in the degradation of organic dyes.

No.	Catalyst	Irradiation Type	Dyes	Removal Ratio (%)	Time (min)	Dye (mg/L)	Catalyst (g/L)	Refs.
1	WO_x_/TiO_2_	visible light	MO	83.0	300	40	1.0	[14]
2	WO_3_/BiOCl	visible light	RhB	100	180	30	0.5	[22]
3	BiVO_4_/TiO_2_/GO	visible light	RB-19	95.87	90	0.05	0.6	[29]
4	BiOCl/TiO_2_-zeolite	visible light	RhB	~100	90	10	1.0	[43]
5	BiOCl/TiO_2_	visible light	RhB	~100	35	20	1.0	[37]
6	RGO/BiOCl/TiO_2_	visible light	MB	98.2	40	30	1.0	In this study
7	RGO/BiOCl/TiO_2_	visible light	AB-10B	96.0	10	30	1.0	In this study
8	RGO/BiOCl/TiO_2_	visible light	MO	98.3	15	30	1.0	In this study
9	RGO/BiOCl/TiO_2_	visible light	RhB	90.5	5	30	1.0	In this study

## Supplementary Materials

The following are available online at https://www.mdpi.com/1996-1944/13/11/2529/s1, Figure S1: (a) XPS wide scan spectrum of GO; (b,c) XPS high resolution spectra of GO: (b) C 1s and (c) O 1s; Figure S2: SEM images of GO.
