# Supplementary material for: Novel Ternary Heterogeneous Reduction Graphene Oxide (RGO)/BiOCl/TiO2 Nanocomposites for Enhanced Adsorption and Visible-Light Induced Photocatalytic Activity toward Organic Contaminants
Source: Materials (Basel). 2020 Jun 2;13(11):2529. doi: 10.3390/ma13112529 (PMC7321430; doi:10.3390/ma13112529)
Supplement: Supplementary file 1 [file materials-13-02529-s001.pdf]

*materials*

*Supplementary Information*

# Novel Ternary Heterogeneous Reduction Graphene Oxide (RGO)/BiOCl/$TiO_2$ Nanocomposites for Enhanced Adsorption and Visible-Light Induced Photocatalytic Activity Toward Organic Contaminants

**Zhanxin Jing *, Xiangyi Dai, Xueying Xian, Qiongshan Zhang, Huojiao Zhong and Yong Li ***

College of Chemistry and Environment, Guangdong Ocean University, Zhanjiang, Guangdong 524088, China; 18813497996@163.com (X.D.); xxy970901@163.com (X.X.); 13692360522@163.com (Q.Z.); 15015931358@163.com (H.Z.)

*Correspondence: jingzhan_xin@gdou.edu.cn (Z.J.); liyong@gdou.edu.cn (Y.L.); Tel.: +86-0759-238-3300 (Z.J.)

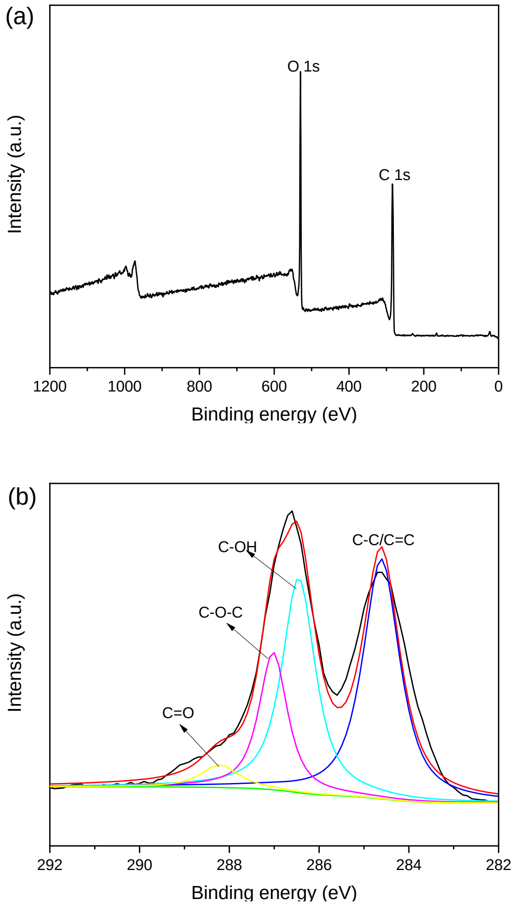

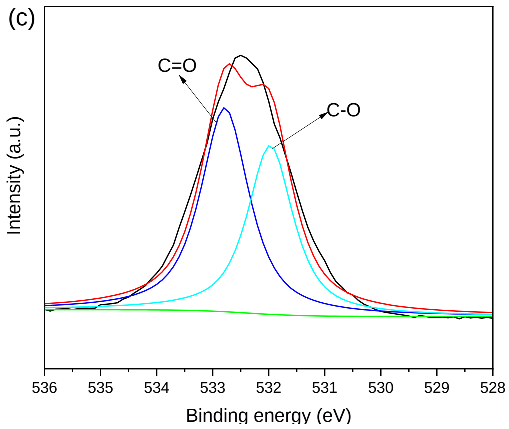


**Figure S1.** (**a**) XPS wide scan spectrum of GO; (**b**, **c**) XPS high resolution spectra of GO: (**b**) C 1s and (**c**) O 1s.

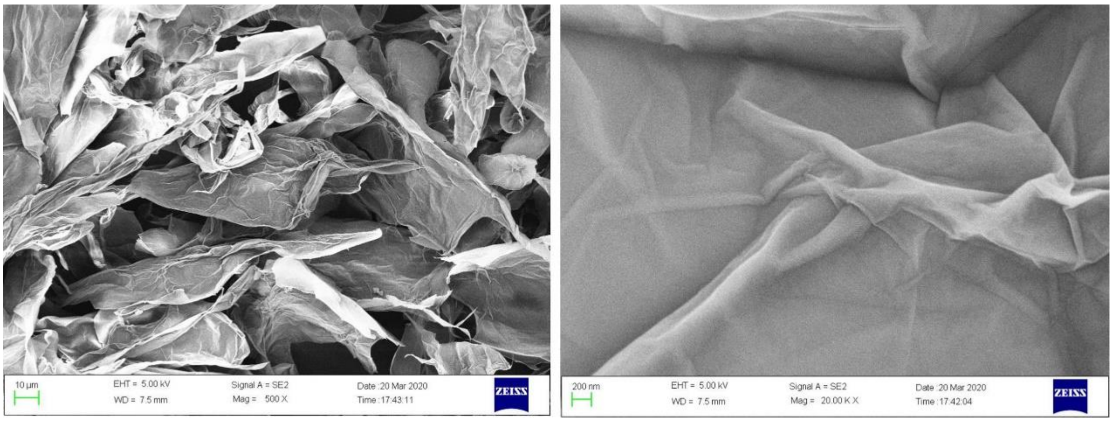


**Figure S2.** SEM images of GO.
